# Gut symbiont enhances insecticide resistance in a significant pest, the oriental fruit fly *Bactrocera dorsalis* (Hendel)

**DOI:** 10.1186/s40168-017-0236-z

**Published:** 2017-02-01

**Authors:** Daifeng Cheng, Zijun Guo, Markus Riegler, Zhiyong Xi, Guangwen Liang, Yijuan Xu

**Affiliations:** 1 Department of Entomology, South China Agricultural University, Guangzhou, 510640 China; 2 Hawkesbury Institute for the Environment, Western Sydney University, Penrith, NSW 2751 Australia; 3 Sun Yat-sen University—Michigan State University Joint Center of Vector Control for Tropical Diseases, Guangzhou, Guangdong 510080 China; 4 Department of Microbiology and Molecular Genetics, Michigan State University, East Lansing, MI 48824 USA

**Keywords:** Symbiotic bacteria, Insecticide resistance, Trichlorphon, *Bactrocera dorsalis*, Oriental fruit fly

## Abstract

**Background:**

Symbiotic bacteria affect insect physiology and ecology. They may also mediate insecticide resistance within their hosts and thereby impact pest and vector control practices. Here, we document a novel mechanism of insecticide resistance in which a gut symbiont of the tephritid pest fruit fly *Bactrocera dorsalis* enhances resistance to the organophosphate insecticide trichlorphon.

**Results:**

We demonstrated that the gut symbiont *Citrobacter* sp. (CF-BD) plays a key role in the degradation of trichlorphon. Based on a comparative genomics analysis with other *Citrobacter* species, phosphatase hydrolase genes were identified in CF-BD. These CF-BD genes had higher expression when trichlorphon was present. *Bactrocera dorsalis* inoculated with isolated CF-BD obtained higher trichlorphon resistance, while antibiotic-treated flies were less resistant confirming the key role of CF-BD in insecticide resistance.

**Conclusions:**

Our findings suggest that symbiont-mediated insecticide resistance can readily develop in *B. dorsalis* and may represent a more widely relevant insecticide resistance mechanism than previously recognized.

**Electronic supplementary material:**

The online version of this article (doi:10.1186/s40168-017-0236-z) contains supplementary material, which is available to authorized users.

## Background

Insects can possess symbiotic microorganisms in their gut lumen, in specialized organs, or within cells [1–4]. In general, such microbial partners can contribute to the nutrition of various insect groups [5], defense against natural enemies [6], reproductive traits [7], and other physiological and ecological properties of insects [8–12]. Some symbiotic bacteria also mediate detoxification of insect diets [13–16] and, similarly, of insecticides, therefore conferring insecticide resistance to their hosts as it has originally been discovered for the apple maggot *Rhagoletis pomonella* [17] and more recently demonstrated for stinkbugs [18, 19].

Chemical insecticides have been widely used to control insect pests and vectors [20]; however, many insect pests and vectors have evolved strong resistance to a diverse range of insecticides. The mechanisms underlying insecticide resistance vary across pesticides and include changes of drug target sites, increased expression of degrading enzymes, and enhanced drug excretion [21, 22]. The frequent failure of chemical control has globally drawn major research attention to resistance mechanisms and management. For example, it has been determined that certain bacteria also possess the ability to degrade pesticides [23, 24], suggesting that symbiotic bacteria of insects may also contribute to insecticide resistance. However, besides the examples of *R. pomonella* [17] and stinkbugs [18], it is not known whether bacterially facilitated insecticide resistance also occurs in other insect pest taxa of economic significance and, further, what the general mechanisms of symbiont-facilitated insecticide resistance are.

Previous studies have found that intensive insecticide application can accelerate insecticide biodegradation in the environment [25, 26], including by bacteria that are capable of degrading organophosphorus compounds [27]. Studies have found that the biochemistry of organophosphorus compound degradation is identical in most bacteria. The functional enzyme in this process, organophosphate hydrolase or phosphotriesterase, is an organophosphate hydrolase encoded by the *opd* (organophosphate-degrading) gene, which has been isolated from taxonomically different bacterial species and from various geographical regions [28].

The oriental fruit fly *Bactrocera dorsalis* (Hendel) (Diptera: Tephritidae) is a significant pest species that damages a wide range of fruit and other horticultural products [29, 30], causing major financial losses to horticulture [31]. Trichlorphon [dimethyl (2, 2, 2-trichloro-1-hydroxyethyl) phosphate] is a moderately toxic organophosphate insecticide that has been widely used to control this pest because of its low toxicity to humans and its high efficacy; however, resistance to this pesticide in *B. dorsalis* has been increasing [32, 33], thus threatening the effective management of the oriental fruit fly.

Several studies have been performed to elucidate the mechanism of *B. dorsalis* resistance to trichlorphon [33, 34]. The functional proteins involved in the resistance response to trichlorphon were identified based on proteomic analyses of *B. dorsalis* treated with trichlorphon [34]. Independent from this, some pesticide-degrading bacteria have previously been isolated from trichlorphon-contaminated soil [35]. We therefore hypothesized that symbiotic bacteria of *B. dorsalis* could increase its resistance to chemical insecticides by degrading trichlorphon. Here, we compared the diversity and abundance of the gut bacteria of *B. dorsalis* strains with different resistance levels in order to identify any changes in their bacterial community composition. We then isolated and cultivated the gut bacteria that were more prevalent in resistant *B. dorsalis* lines and obtained one bacterium, *Citrobacter freundii* BD (CF-BD) that was able to degrade trichlorphon. We then manipulated insecticide resistance by adding or removing CF-BD and provided further evidence for a close relationship between this bacterium and the pesticide resistance in *B. dorsalis*. Based on the annotated genome sequence of the isolated bacterium, together with a comparative genomics analysis, new phosphatase genes were identified in CF-BD when compared with other *Citrobacter* species and their expression studied when exposed to trichlorphon.

## Results

### Trichlorphon-resistant and trichlorphon-susceptible fly strains exhibit different gut symbiotic bacterial communities

**Fig. 1:**
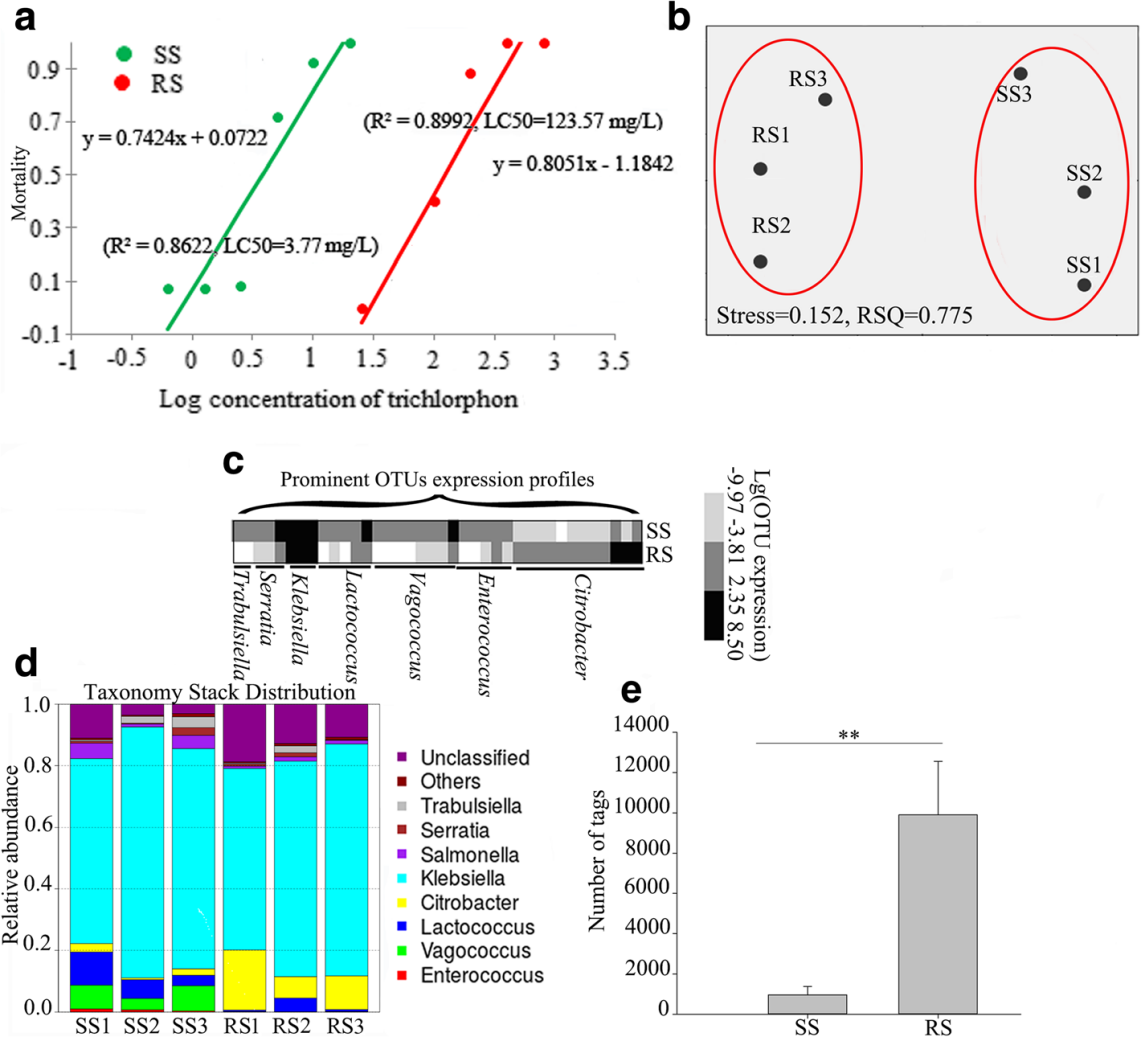
Different symbiotic bacterial communities in the gut of susceptible and resistant strains of *Bactrocera dorsalis*. **a** Toxicity regression analyses of susceptible (SS; *green line*) and resistant (RS; *red line*) fly responses to trichlorphon. **b** Non-metric multidimensional scaling (nMDS) plot exhibiting a structural difference (according to the Bray-Curtis index) between the bacterial communities of SS and RS. **c** A heat map reflecting the mean number of the prominent OTUs in SS and RS. The data were log-transformed before plotting. **d** A stack map at the genus level for six samples. **e** Differences in the numbers (mean ± standard error (SE)) of tags that were assigned to *Citrobacter* between SS and RS. Statistically significant differences at the *p* < 0.01 level, as evaluated with independent-sample *t* tests are indicated by *two asterisks above the bars*

To confirm the resistance levels of the *B. dorsalis* susceptible (SS) and resistant (RS) strains, the toxicity of ingested trichlorphon was examined; the dose-response curves are shown in Fig. 1a. The median lethal concentration (LC_50_) of trichlorphon was 3.77 mg/L for SS and 123.57 mg/L for RS.

To assess the diversity of the gut symbiotic bacteria of SS and RS, the variable region of the 16S rDNA was sequenced via high-throughput amplicon sequencing. By filtering sequence reads, tags were generated for the six samples analyzed. As shown in Additional file 1: Table S1, the greatest number of tags was observed in RS1, and the fewest tags were found in SS3. OTUs were successfully generated for the six samples; as shown in Additional file 2: Table S2, Additional file 3: Table S3 produced the greatest number of OTUs. OTU abundance profiles were obtained for all of the samples by combining the OTU species annotation information and abundance information across different samples.

Specifically, more than 81.25% of the sequence reads were successfully annotated to the genus level, although less than 1.42% of the reads were annotated to the species level. The structure of bacterial communities in RS differed markedly from those in SS (Fig. 1b, c). A stack map at the genus level for the six samples demonstrates that the number of reads assigned to *Citrobacter* was significantly greater among RS than SS (independent-sample *t* test, *t* = 3.354, df = 4, *p* = 0.028; Fig. 1d, e).

### *Citrobacter* sp. isolation and identification

**Fig. 2:**
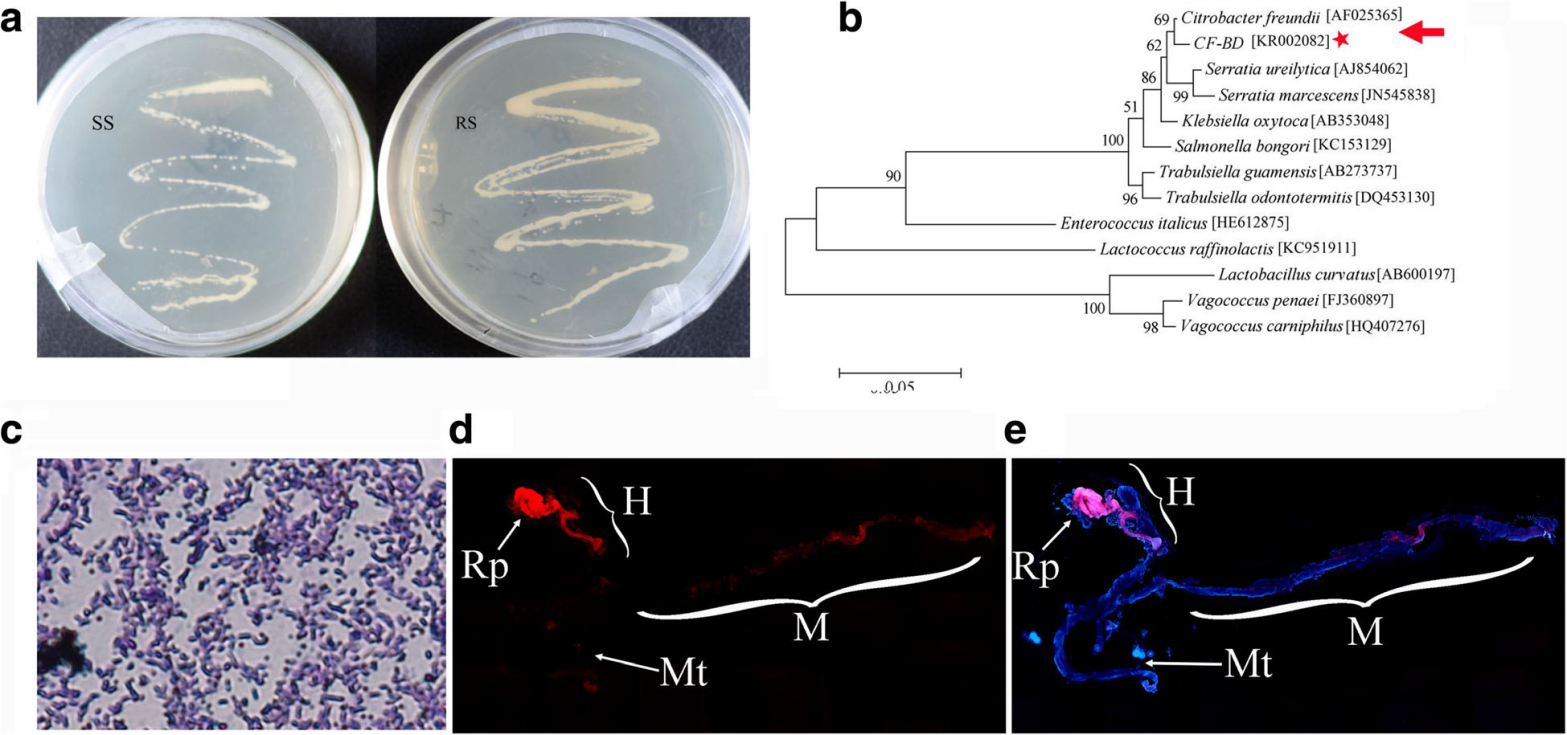
*Citrobacter* sp. isolation and identification. **a** Colony characteristics on brain heart infusion (BHI) agar plates for bacteria that were isolated from the gut of SS and RS flies. **b** Phylogenetic relationships of the symbiotic CF-BD strain. The *red star* indicates the trichlorphon-degrading CF-BD strain. A maximum likelihood phylogeny inferred from 1521 aligned nucleotide sites in 16S rDNA gene sequences is presented with bootstrap values. The accession numbers of the 16S rDNA sequences for each bacterium are listed in the *square brackets*. **c** The rod-shaped CF-BD strain has blunt, round ends and a *red* color, as identified by Gram staining. **d**, **e** The midgut organization of *B. dorsalis* and CF-BD localization in the midgut of RS flies. *Red* signals indicate CF-BD symbionts, whereas *blue* signals show host insect nuclei. Abbreviations: M, midgut; Mt, Malpighian tubules; H, hindgut; Rp, rectal pads

In order to isolate *Citrobacter* sp., subculturing was performed on BHI agar flat plates, and the bacteria were identified by 16S rDNA sequencing and analyses of their physiological and biochemical characteristics. After 24 h, *Citrobacter* sp. colonies grown from the SS and RS isolates were identified by a white color, raised center, clean margin, and smooth, wet surface (Fig. 2a). 16S rDNA amplification and sequencing yielded a fragment of 1521 bp. Based on a BLAST search against GenBank, the 16S rDNA sequence exhibited 99% identity with *Citrobacter freundii*, and we named the strain CF-BD (Fig. 2b). The sequence was deposited in GenBank (accession number KR002082). Identification using the GYZ-15 eV system also indicated that CF-BD was most similar with *Citrobacter freundii* (Table 1). The Gram stain test was negative, and morphological analysis revealed that the bacterium was a short rod of approximately 0.5–0.9 μm × 1.2–2.0 μm with blunt, round ends (Fig. 2c).

**Table 1:** Identification of bacterial BD-1 by the GYZ-15eV system

Characteristics	BD-1	*C. freundii*
Xylose	+	+
Sorbitol	+	+
Adonitol	−	−
Carbamide	−	−
Simmons citrate	+	+
Gluconate	−	−
Phenylalanine	−	−
Reffinose	+	+
Peptone water	−	−
Dextrose Phosphate Peptone Water	+	+
Ornithine decarboxylase	+	+
Lysine decarboxylase	−	−
Sulphureted hydrogen	+	+
Semi-solid agar	−	−
Triple-Sugar Iron Agar	+	+

“+” positive; “-”negative

### In vivo localization of CF-BD symbionts

The gut of *B. dorsalis* was dissected and subjected to fluorescence in situ hybridization (FISH) targeting 16S rRNA of CF-BD symbionts. CF-BD signals were consistently localized in the midgut crypts of the whole gut of *B. dorsalis* (Fig. 2d, e).

### Detection of CF-BD symbionts from diverse populations

**Fig. 3:**
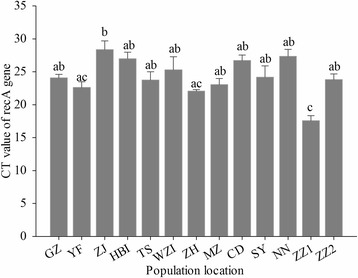
Detection of CF-BD in *Bactrocera dorsalis* from diverse populations. *GZ* Guangzhou, *YF* Yunfu, *ZJ* Zhanjiang, *HBI* Hebao island, *TS* Taishan, *WZI* Weizhou island, *ZH* Zhuhai, *MZ* Mengzi, *CD* Chengdu, *SY* Sanya, *NN* Nanning, *ZZ1* Zhengzhou, and *ZZ2* Zhangzhou. *Bars* (mean ± SE) labeled with the same letter within each treatment are not significantly different (*p* > 0.05, Tukey’s test)

Based on the genome sequence information, a primer pair for *recA* PCR amplification was designed to screen for CF-BD infections in flies. Flies from wild populations were subjected to diagnostic and quantitative PCR. As a result, CF-BD was detected in all 78 individuals from 13 populations, with 100% infection. However, individuals from different populations differed significantly in their CF-BD densities. Flies from Zhengzhou, Henan, had the highest density of CF-BD, and flies from Zhanjiang, Guangdong, had the lowest density of CF-BD (*F* = 6.38, df = 12, *p* < 0.01, Fig. 3).

### The insecticide resistance of RS flies is decreased by CF-BD deprivation

**Fig. 4:**
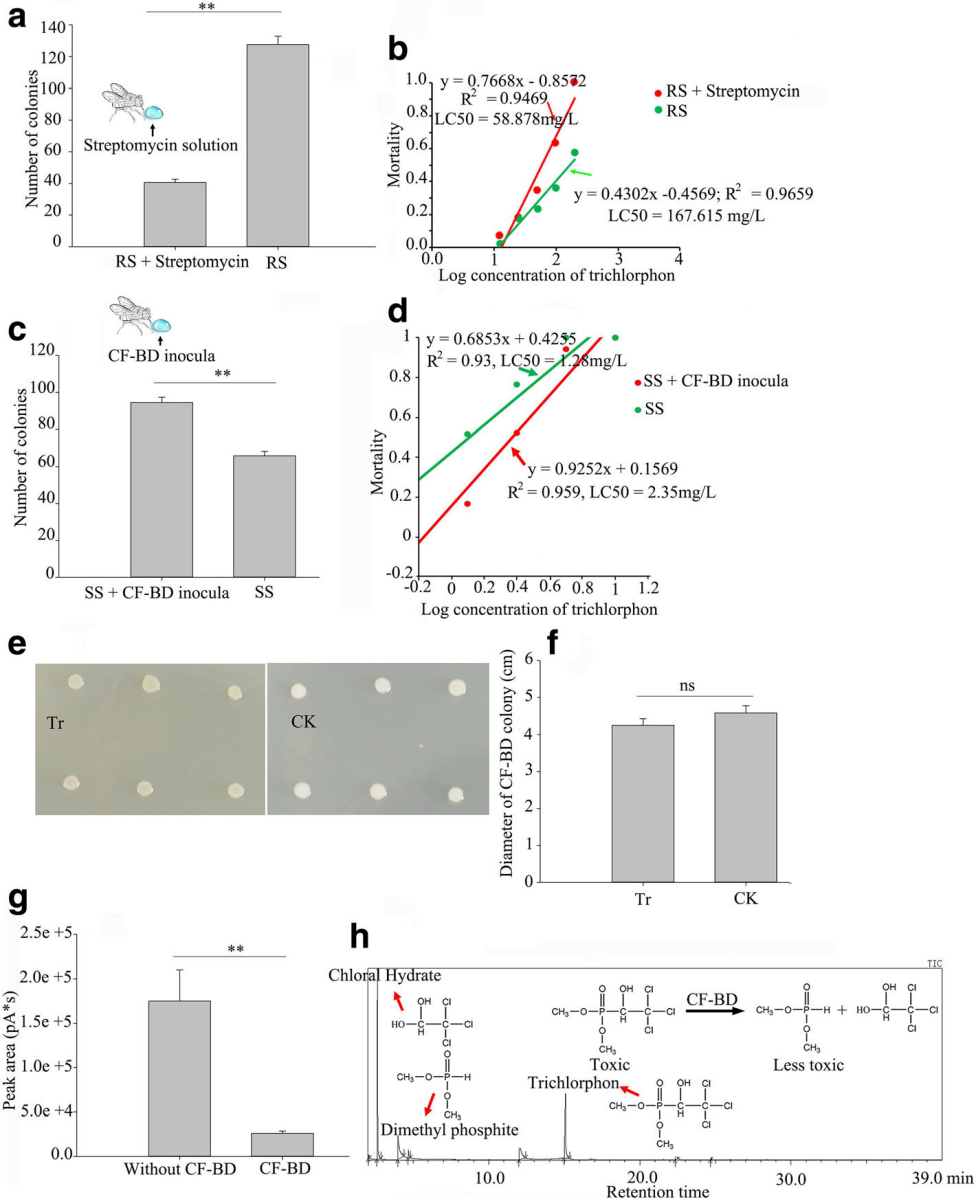
Trichlorphon tolerance and degradation ability of CF-BD and its effects on the drug susceptibility and resistance of flies. **a** A comparison of the number of CF-BD colony forming units (mean ± SE) between the RS flies and those that were given streptomycin. **b** Toxicity regression analyses of the RS (*red line*) and streptomycin-fed RS (*green line*) fly responses to trichlorphon. **c** A comparison of CF-BD colony numbers (mean ± SE) between SS flies and CF-BD-fed SS flies. **d** Toxicity regression analyses of of SS (*green line*) and CF-BD-fed SS (*red line*) fly responses to trichlorphon. **e** Characteristics of bacteria that were cultivated on trichlorphon-enriched plates and normal plates. **f** Diameters of the bacterial colonies (mean ± SE) cultivated on trichlorphon-enriched plates and normal plates. **g** The gas chromatograph identification of trichlorphon in the filtrate of mineral media with or without CF-BD cultures. Differences in the trichlorphon concentrations were evaluated by comparing the peak areas (mean ± SE) that were identified by gas chromatography. **h** The gas chromatography mass spectrometry (GC-MS) identification of the trichlorphon degradation products of CF-BD (the degradation pathway is presented). The significant differences at the *p* < 0.01 level, as evaluated with independent-sample *t* tests, are indicated by *two asterisks above the bars. ns* indicates not significant. *Tr* trichlorphon-enriched plates, *CK* control plates

Antibiotic sensitivity tests revealed that CF-BD is highly sensitive to streptomycin, sulfisoxazole, sulfamethoxazole, chloramphenicol, ciprofloxacin, tetracycline, and nalidixic acid. However, CF-BD was resistant to amikacin, ampicillin, cefazolin, and amoxicillin (Additional file 4: Table S4). Thus, we used streptomycin to clear CF-BD from the guts of RS flies. After feeding with streptomycin, the number of CF-BD colonies obtained from RS fly gut samples was significantly reduced compared with the RS gut samples from flies that were fed sterile water (independent-sample *t* test, *t* = 15.496, df = 8, *p* < 0.01; Fig. 4a). This indicated that the abundance of CF-BD in the fly gut was significantly decreased after feeding with an antibiotic solution. Moreover, the RS flies that were fed the streptomycin solution were more sensitive to trichlorphon than the RS flies (two-way ANOVA *F* = 66.905, df = 1, *p* < 0.0001; LC_50_ = 89.78 mg/L for RS+ streptomycin, LC_50_ = 133.31 mg/L for RS; Fig. 4b), indicating that CF-BD was required for enhanced resistance to trichlorphon.

### The insecticide resistance of SS flies is increased after CF-BD supplementation

We also attempted to increase the abundance of CF-BD in the guts of SS flies by feeding them with CF-BD inoculum. After feeding SS flies CF-BD, the amount of gut CF-BD was significantly higher than in the control flies that were given water (independent-sample *t* test, *t* = 7.67, df = 8, *p* < 0.01; Fig. 4c). Moreover, the SS flies that were fed the CF-BD inoculum were more resistant to trichlorphon than the control flies (two-way ANOVA *F* = 7.848, df = 1, *p* = 0.011; LC50 = 2.35 mg/L for SS+ CF-BD inocula, LC_50_ = 1.28 mg/L for SS; Fig. 4d), which indicated that increases in CF-BD in the guts of flies increased their resistance to trichlorphon.

### Trichlorphon tolerance and degradation ability of CF-BD

To investigate the function of CF-BD in relation to trichlorphon resistance, the trichlorphon tolerance and degradation ability of CF-BD was tested by adding CF-BD to trichlorphon-enriched BHI agar plates. As the growth rates and diameters of the colonies did not differ between the trichlorphon-enriched and non-enriched plates (independent-sample *t* test, *t* = 1.247, df = 10, *p* = 0.241), CF-BD was not inhibited by trichlorphon (Fig. 4e, f). Gas chromatography analysis revealed a greater amount of trichlorphon in the filtrate purified from the trichlorphon-enriched medium without CF-BD (Fig. 4g, independent-sample *t* test, *t* = 4.217, df = 4, *p* = 0.014), which suggests that trichlorphon was degraded by the bacterium. Furthermore, GC-MS analysis revealed that trichlorphon was degraded into chloral hydrate and dimethyl phosphite, which are significantly less toxic than trichlorphon [36, 37] (Fig. 4h).

### Predicting trichlorphon degradation ability through a genome analysis of CF-BD

**Fig. 5:**
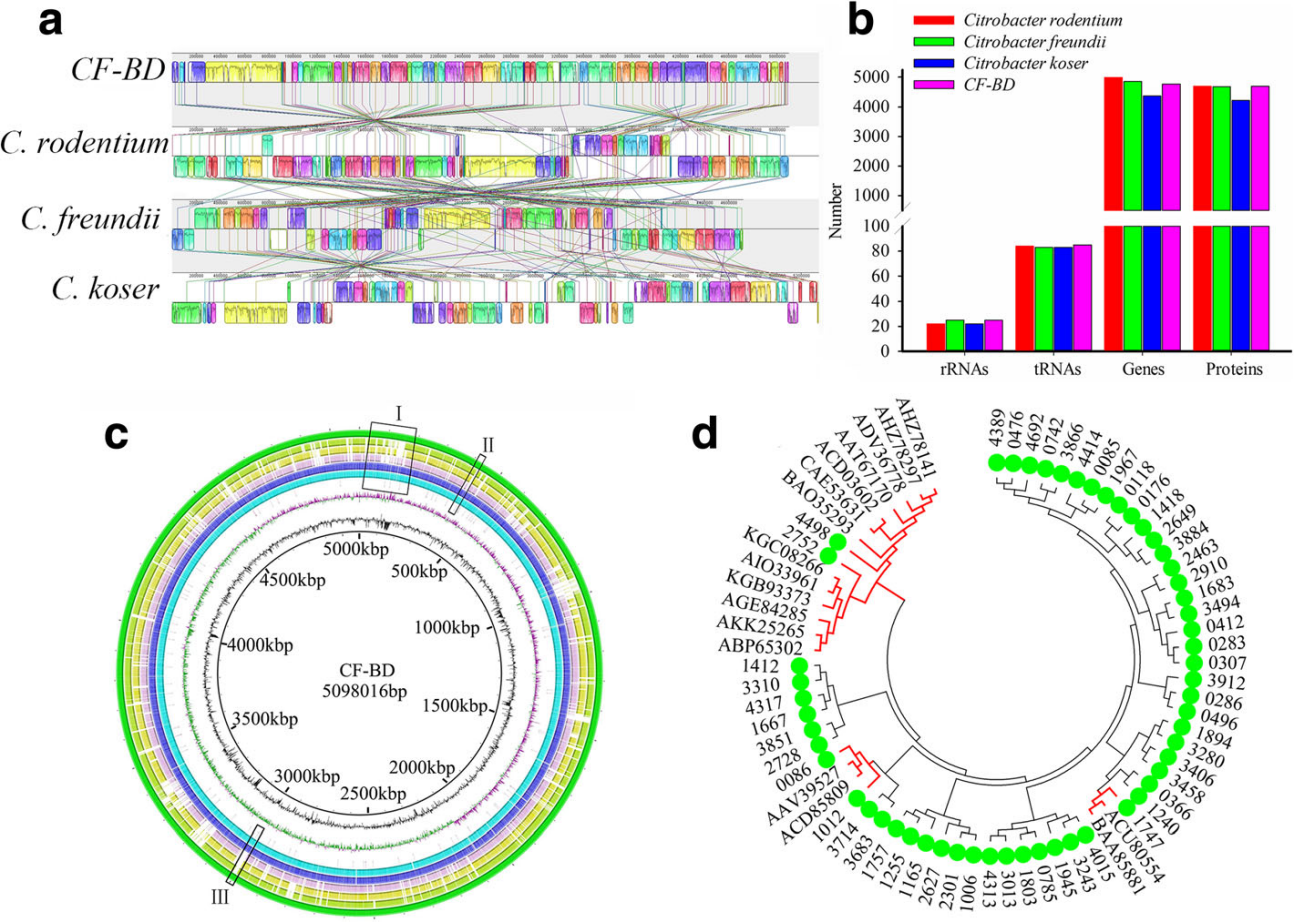
CF-BD genomic characterization and comparative analysis. **a** Synteny analysis between the genome of *Citrobacter freundii*, *Citrobacter koseri*, *Citrobacter rodentium*, and CF-BD. **b** Genomic components comparison between *C. freundii*, *C. koseri*, *C. rodentium*, and CF-BD. **c** Alignment of the CF-BD genome (*circle 1* from the outside in) with *C. freundii* (*circle 2*), *C. koseri* (*circle 3*), and *C. rodentium* (*circle 4*) by applying BLAST and BLAST Ring Image Generator (BRIG). Similarity is symbolized by *colored blocks*. The more intense the color, the higher the similarity. CF-BD-specific regions are highlighted by *black frames* labeled with Roman numerals (*I*–*III*). *Circles 5*, *6*, and *7* represent the predicted coding DNA sequence (CDS), the ORF of the function-annotated genes, and the phosphatase genes, respectively. *Circles 8* and *9* represent the GC skew and GC content, respectively. **d** The phylogenetic relationship of the different phosphate hydrolase families. Family members with *green dots* are from CF-BD and others are organophosphorus hydrolase genes of other bacteria. The *red trees* indicate potential OPH genes in CF-BD (accession numbers for genes are listed in Additional file 8: Table S6)

To predict the CF-BD trichlorphon degradation ability, the CF-BD genome was further analyzed. The draft genome of CF-BD contains one scaffold with 5,098,016 bp, 4762 predicted coding sequences (CDS), and 25 rRNA and 85 tRNA genes. Putative functions were assigned to 4689 of the predicted CDS. The average G + C content was 52.06%. Among the function-annotated genes, 55 phosphatase genes were identified. Comparative genomics that employed the sequences of the fully available genomes of *C. rodentium*, *C. koseri*, *C. freundii*, and CF-BD revealed large clusters that were only found in CF-BD (Fig. 5a, c). Also, the genomic components of CF-BD were different from those of *C. rodentium*, *C. koseri*, and *C. freundii* (Fig. 5b). In particular, the clusters highlighted at positions I, II, and III contained five phosphatase genes (gene ID 0086, 1012, 1747, 2752. and 4498) that were specifically present in CF-BD (Fig. 5c). Analysis of sequence similarities suggested that these five phosphatase genes were more similar to the organophosphorus hydrolase genes (OPH) of other bacteria (Fig. 5d), which assigned potential function to degrade the organophosphorus to the five genes.

**Fig. 6:**
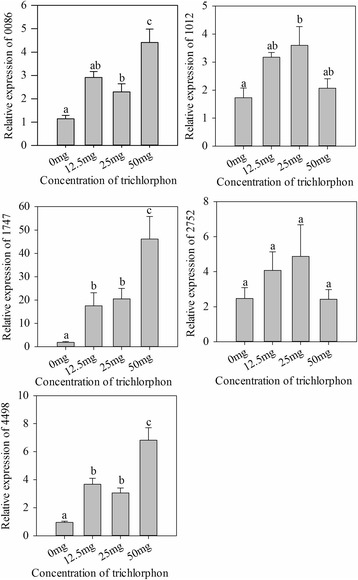
Relative expression of the five OPH-like genes stimulated by trichlorphon. Expression of OPH genes was measured against the reference gene *recA. Bars* (mean ± SE) labeled with the same *letter* within each treatment are not significantly different (*p* > 0.05, Tukey’s test)

In order to verify the function of the five identified OPH-like genes, the expression of these genes was measured with the CF-BD exposed to trichlorphon at different concentrations. The results showed the expression of genes 0086, 1012, 1747, and 4498 were significantly higher when exposed to trichlorphon (0086 *F* = 13.995, df = 34, *p* < 0.001; 1012 *F* = 4.36, df = 34, *p* = 0.011; 1747 *F* = 9.291, df = 34, *p* < 0.001; 4498 *F* = 21.092, df = 34, *p* < 0.001; Fig. 6).

## Discussion

We report the first isolation of *Citrobacter freundii* strain (CF-BD) associated with trichlorphon resistance from the digestive tract of the significant pest fruit fly *B. dorsalis*. Using 16S rDNA and genome sequencing, Gram staining, physiological and biochemical identification procedures, and fluorescence in situ hybridization, we confirmed that some of the bacteria residing in the midgut crypts of the fly are similar to *C. freundii* and enhance the fly’s insecticide resistance. With the advent of sequencing technology, many bacterial communities have been identified in the gut of various insects [38–40], including of tephritid fruit flies [11, 41, 42]. *Citrobacter* is a commonly found symbiotic taxon in insects and fruit flies specifically [43] and belongs to the Gammaproteobacteria, a class that includes dominant symbiotic bacteria in many insect lineages [44, 45]. We determined that the dominant symbiotic bacteria in the gut of oriental fruit flies were in the family of Enterobacteriaceae and, in particular, in the genus *Klebsiella* (Fig. 1d), suggesting that these bacterial symbionts may play a role in the biology of this fly species. CF-BD was also present in all flies at variable and lower densities. *Citrobacter freundii* includes pathogenic isolates that can cause respiratory and urinary tract infections in humans [46]; strains of this species can also be pathogenic to fish [47]. In contrast, oriental fruit flies do not exhibit pathology associated with CF-BD, and an increased titer of this bacterium was found in the insecticide resistant strain (RS), indicating a different function of this bacterium in the gut of resistant versus susceptible flies. Moreover, several genomic differences were found between CF-BD and other *C. freundii* strains, suggesting that CF-BD is not necessarily pathogenic to humans or fish.

Growth on trichlorphon-enriched plates and the degradation of trichlorphon in the mineral medium demonstrated that CF-BD can degrade trichlorphon (Fig. 4e–h). So far, many bacterial species have been shown to possess this ability, although these bacteria were largely isolated from environmental samples such as from soil [28, 48]. We determined that CF-BD isolated from fruit flies was able to degrade trichlorphon and enhance the resistance of the flies to trichlorphon. Similar results have been obtained for a bacterium isolated from *R. pomonella* [17] and stinkbugs, in particular, the bean bug *Riptortus pedestris* [18]. In *R. pedestris*, resistance has been observed with both oral and percutaneous applications of the insecticide. In this stinkbug species, gut symbiotic bacteria of the genus *Burkholderia* can be acquired by nymphs from soil. While our study demonstrated that CF-BD in *B. dorsalis* enhances resistance to trichlorphon, it remains unknown how CF-BD is transmitted through life stages and in fly populations. Our field survey detected CF-BD at low densities in all tested adult flies; however, it is not clear whether CF-BD is maternally inherited, horizontally transmitted between larvae or adults, or taken up from the environment.

Microbial symbionts of insect species can have important functions in their hosts. In bark beetle, symbiotic bacteria can supply essential nutrients and assist with digestion and detoxification of plant compounds [49], and this was also seen in the coffee berry borer [16]. In aphids, *Hamiltonella defensa* [50] can increase resistance to hymenopteran parasitoids [6, 51] via toxin-encoding bacteriophages [52] that kill parasitoid eggs or early larvae. Some microbial symbionts are able to assist the host in escaping predation [53] and increase host resistance to *Bacillus thuringiensis* [54]. Furthermore, microorganisms present in the gut can provide additional functions, including the digestion of plant polymers and the detoxification of plant-produced toxins [55]. In bean bugs, *Burkholderia* was rapidly established within a single insect generation after environmental acquisition and shared horizontally between different individuals [18]. Although we have demonstrated that CF-BD can be obtained by oral feeding, additional evidence is needed to determine whether it can be transmitted horizontally in a manner similar to *Burkholderia*.

Many organophosphate pesticide-degrading bacteria have been reported in recent years [48, 56, 57]. Moreover, many *Citrobacter* species can degrade phenols and phosphates [58–60], and their degradation pathways are widely recognized. Myo-inositol hexakisphosphate can be degraded by a phytase from *Citrobacter braakii* [58], and the degradation of *m*-cresol via the *ortho* cleavage pathway by *Citrobacter farmeri* SC01 has also been reported [59]. In our study, we isolated CF-BD from the gut of *B. dorsalis*, although its organophosphate-degrading ability has previously not been reported. Genome sequence analysis of CF-BD revealed 55 phosphatase genes, and within these, five new genes were unique to CF-BD. Sequence similarities of the five genes revealed that they were OPH-like, and their expression was stimulated by the presence of trichlorphon. We also found that trichlorphon was degraded into chloral hydrate and dimethyl phosphite by CF-BD. Although the characteristics of these metabolites are extremely similar to results obtained with *C. braakii*, additional evidence is needed to determine whether this degradation pathway is the same as that utilized by *C. braakii* and whether phosphatase is the critical enzyme in this pathway. We only sequenced the *C. freundii* genome in RS flies, which were collected in 2008 and then experienced serial selection for insecticide resistance in the laboratory. Whether the CF-BD genome has changed between the field collection and after selection for insecticide resistance remains unknown. *Citrobacter freundii* in SS flies may have lower insecticide degradation capacity than CF-BD of RS flies, and future research should address this, in combination with comparative genomics approaches. Until this has been tested further, we conclude that the enhanced insecticide resistance in *B. dorsalis* in our study is due to increased abundance of CF-BD in the gut.

In this study, we isolated a bacterium with an important role in the resistance of *B. dorsalis* to trichlorphon, and this symbiont was widespread in the wild population of the flies (Fig. 3); thus, regulation of this symbiont might be useful for managing trichlorphon resistance. Moreover, our findings also demonstrate the necessity of considering the gut symbiotic bacteria of insects during the implementation of novel pest control measures [61]. Because the generation times of bacteria are considerably shorter than those of the host insects, the evolution of insecticide resistance in insects may proceed much more rapidly via symbiont-mediated processes.

## Conclusions

We document a novel mechanism of insecticide resistance in which a gut bacterium (*Citrobacter freundii*) of *Bactrocera dorsalis* enhances the fruit flies’ resistance to trichlorphon, and we experimentally demonstrated that *C. freundii* degrades trichlorphon into chloral hydrate and dimethyl phosphite. These results indicate that symbiont-mediated trichlorphon resistance might develop in oriental fruit flies.

## Methods

### Insects

The susceptible strain (SS) of *B. dorsalis* was collected from a carambola (*Averrhoa carambola*) orchard in Guangzhou, Guangdong Province, in April 2008, and was maintained in the laboratory for approximately 80 generations under the following conditions: 25 ± 1 °C; 16:8 h light:dark cycle; 70–80% relative humidity (RH); a maize-based artificial larval diet containing 150 g corn flour, 150 g banana, 0.6 g sodium benzoate, 30 g yeast, 30 g sucrose, 30 g paper towel, 1.2 mL hydrochloric acid and 300 mL water, and an adult diet consisting of water, yeast hydrolysate, and sugar. Pesticide exposure was avoided during rearing.

A resistant strain (RS) of *B. dorsalis* was obtained by selection after adult exposure to a trichlorphon treated surface over the course of 33 generations. Trichlorphon was diluted with acetone to LC_50_ for each generation and evenly coated onto the inside of a 250-mL conical flask by shaking. After the acetone had volatilized, 30 3–5-day-old fly pairs were placed in the flask for 24 h. The surviving flies were selected for breeding of the next generation [62]. The resistance levels were calculated based on Benson’s formula [63].

### Fly toxicity assay

A total of 15 3–5-day-old mixed (female and male) adult flies were placed in plastic cups that had six intruding 200-μL pipette tips on the top and ventilation holes on the sides (Additional file 5: Figure S1). The tips contained aqueous trichlorphon solutions at different concentrations: 0 mg/L, 0.625 × 10^−^ mg/L, 1.25 × 10^−3^ mg/L, 2.5 × 10^−3^ mg/L, 5 × 10^−3^ mg/L, 10 × 10^−3^ mg/L, and 20 × 10^−3^ mg/L for SS; 0 mg/L, 25 × 10^−3^ mg/L, 50 × 10^−3^ mg/L, 100 × 10^−3^ mg/L, 200 × 10^−3^ mg/L, 400 × 10^−3^ mg/L, and 800 × 10^−3^ mg/L for RS. Three replicate cups with six tips each were prepared for each concentration. All of the treatments were maintained at a temperature of 25 ± 1 °C under a 16:8 h light:dark cycle. Mortality was recorded after 24 h.

### Extraction of DNA from SS and RS fly guts

Three-to-five-day-old mixed (female and male) adult flies were selected and soaked in absolute ethanol for 3 min. The soaked flies were dissected under a stereo-microscope, and the guts were transferred into centrifuge tubes containing DNA extraction buffer. For each sample, 15 flies were dissected, and three samples were collected for both the SS (SS1, SS2, SS3) and the RS (RS1, RS2, RS3) strains. Total DNA of the dissected gut samples was extracted using a DNA extraction kit (Tiangen, Beijing, China) following the manufacturer’s instructions.

### V3 + V4 region of 16S rDNA amplification and sequencing

Approximately 465 bp of the V3 + V4 region of the bacterial 16S rDNA gene was amplified by PCR according to a standard protocol (Additional file 6: Supplementary methods). And the amplified DNA was sequenced using the Illumina sequencing kit and the Illumina MiSeq sequencer (Illumina, San Diego, CA, USA).

### Bioinformatic analysis of sequencing results

After quantity control (Additional file 6: Supplementary methods), sequence reads were subjected to redundancy treatment with Mothur software [64] to count the number of identical tags. For species annotation, an RDP classifier [65] was used with naïve Bayesian settings; the confidence threshold was set to 0.5. To obtain additional information regarding species diversity composition, we subjected the tags to OTU abundance analysis (Additional file 6: Supplementary methods).

### *Citrobacter* sp. isolation and culture

Six 3–5-day-old adult fruit flies (three flies from each strain) were collected and immediately soaked in 70% ethanol for 3 min to remove surface bacteria. The guts of the flies were dissected and collected in six sterile centrifuge tubes to which 20 μL of sterile water was added. The guts were then ground with sterile grinding pestles, and the fluid was streaked and cultivated for 24 h at 30 °C on brain heart infusion (BHI) agar flat plates, which is the specific medium for cultivation of *Citrobacter* sp. Colonies with the same morphology were selected for subculturing. The pure cultures were inoculated into BHI medium, and the liquid cultures were stored in 25% glycerol solution under −80 °C.

### Bacteria identification by 16S rDNA amplification

16S rDNA of the cultivated bacteria was amplified sequenced (Additional file 6: Supplementary methods). The sequences were subjected to a BLAST search against the NCBI database for sequence homology analysis.

### Physiological and biochemical identification of the bacterium and Gram staining

The physiological and biochemical characteristics of the *Citrobacter* isolates were tested using GYZ-15 eV biochemical detection kits produced by Guangzhou Huankai Microbial Sci. & Tech. Co., Ltd. (Guangzhou, China) according to the manufacturer’s instructions. Gram staining kits produced by Guangzhou Huankai Microbial Sci. & Tech. Co., Ltd. were used to determine bacterial morphology under a light microscope.

### Antibiotic and trichlorphon sensitivity testing

To test bacterial sensitivities to antibiotics, 10-μL inocula were streaked onto BHI agar flat plates to which drug susceptibility test papers were subsequently attached (Table 1). The plates were incubated for 24 h at 30 °C, and the diameters of the inhibition zones were then measured. According to CLSI standards, the cultures were classified as sensitive (S), medium (I), or resistant (R). Plates containing 1 × 10^−3^ mg/L trichlorphon were also prepared to test the sensitivity of the bacteria to trichlorphon with a 10-μL inoculum and an incubation period of 24 h. As a control, plates without trichlorphon were prepared and incubated with inocula. The diameters of colonies were measured after 24 h.

### Resistance of flies inoculated with CF-BD

The bacterial inoculum for flies was prepared by selecting and incubating a colony of CF-BD in BHI medium at 30 °C until an OD600 of 0.8. Next, >300 newly emerged SS flies were fed sugar and bacterial inoculum instead of water for 3 days. After 3 days, the guts of five flies were dissected, ground, and diluted with 200 μL sterile water in 1.5-mL centrifuge tubes. Next, 10 μL of the fluid was plated and cultivated for 12 h, and the numbers of colonies were recorded and compared with the SS flies. Then, cohorts of 15 CF-BD treated 3-day old SS flies each were collected and fed a trichlorphon solution to measure the LC_50_. The tested trichlorphon concentrations were 0 mg/L, 0.625 × 10^−3^ mg/L, 1.25 × 10^−3^ mg/L, 2.5 × 10^−3^ mg/L, 5 × 10^−3^ mg/L, and 10 × 10^−3^ mg/L. After 24 h, the number of dead flies was recorded. As control, newly emerged SS flies were fed pure water and after 3 days, provided trichlorphon solutions in cohorts of 15 flies each. For each trichlorphon concentration, three replicates were performed.

### Resistance of flies cleared of CF-BD

More than 300 newly emerged RS flies were fed a streptomycin solution for 2 days to clear them of bacteria. Afterwards, the guts of five flies were dissected, ground, and diluted with 200 μL of sterile water; the sample was used to cultivate bacteria on plates; the number of colonies was recorded and compared with that of the control. Then, cohorts of 15 RS flies each were fed with a trichlorphon solution, and the number of dead flies was counted after 24 h. The trichlorphon concentrations were 0 mg/L, 25 × 10^−3^ mg/L, 50 × 10^−3^ mg/L, 100 × 10^−3^ mg/L, and 200 × 10^−3^ mg/L. As control, newly emerged RS flies were fed pure water and, after 3 days, provided trichlorphon in cohorts of 15 flies each. For each trichlorphon concentration, three replicates were performed.

### Testing of the trichlorphon degradation characteristics of purified bacteria

Mineral medium (MM) was prepared containing the following salts (g/L): NaCl 1.00, (NH_4_)_2_SO_4_ 1.00, K_2_HPO_4_ 1.50, KH_2_PO_4_ 0.50, and MgSO_4_·7H_2_O 0.50 (pH: 7.0–7.5). The enrichment medium was prepared by adding trichlorphon to MM at a concentration of 100 mg/L. To measure degradation by the isolated bacteria, a 2-mL inoculum was added to MM (100 mL); 2 mL pure water added to the MM (100 mL) was used as the control. Three replicates were prepared for each treatment. The inoculated media were shaken and cultivated at 30 °C for 24 h. Then, 2 mL of the cultures was collected for quantitative analyses of trichlorphon. To purify trichlorphon, 4 mL acetone was added to the culture, which was then shaken for 1 h. NaCl was added until saturation; the solution was shaken for 1 min and allowed to stand for 5 min at room temperature before the acetone layer was collected and filtered through a 45-μm bacterial membrane filter. The filtrate was dried with anhydrous sodium sulfate and used for gas chromatography. The detection conditions were as follows: air flow 5 mL/min, hydrogen flow 5.5 mL/min, carrier gas (nitrogen) flow 8 mL/min, detector temperature 230 °C, injection port temperature 200 °C, chromatographic column temperature 150 °C, and injection volume 0.5 μL. Acetone was used as the solvent, and the peak area was used to quantify trichlorphon. We further examined the filtrate by GC-MS to identify the degradation products.

### Fluorescence in situ hybridization

To locate the CF-BD in the *B. dorsalis* gut, FISH was conducted for RS flies. The oligonucleotide probe called the CFBD-probe (5′-AATGGCGTACACAAAGAG-3′) which was used for in situ hybridization, was labeled with Cy3 at the 5′ end. The probe specifically targeted different regions in the 16S rRNA of the CF-BD symbiont, and it was simultaneously used to enhance the hybridization signals. The dissected gut samples were incubated in a hybridization buffer [20 mM Tris-HCl (pH 8.0), 0.9 M NaCl, 0.01% sodium dodecyl sulfate (SDS), and 30% formamide] containing 50 nM probe. Following overnight incubation, the samples were thoroughly washed in phosphate-buffered saline (PBS) and mounted in SlowFade antifade solution (Molecular Probes, Chuo-ku, Japan). The samples were observed under an epifluorescent microscope (Axiophot, Carl Zeiss, Shinjuku-ku, Japan).

### CF-BD genome sequencing and annotation

To investigate the function of CF-BD at the genomic level, the genome of CF-BD was sequenced. After genome assembly and comparative genome analyses, potential organophosphorus hydrolase genes (OPH) were identified and compared with other similar genes from other bacteria (Additional file 6: Supplementary methods).

### Expression analysis of OPH-like genes by real-time qPCR

To identify the potential function of the OPH-like genes, total RNA of CF-BD cultivated for 20 h in the BHI medium containing 0, 12.5, 25, and 50 mg trichlorphon were extracted to analyze the expression of the OPH-like genes. Complementary DNA (cDNA) was reverse-transcribed from 2 μg total RNA using MMLV reverse transcriptase (Promega). The *recA* gene was used as the reference gene [66]. Real-time quantitative PCR amplification was performed using Mx3000P spectrofluorometric thermal cycler (Agilent Technologies, Santa Clara, CA, USA) and Real Master Mix (SYBR Green) kit (Tiangen), starting with a 2 min incubation at 95 °C, followed by 40 cycles of 95 °C, 20 s; 55 °C, 1 min; and 72 °C, 30 s. Primer information for the genes was described in Additional file 3: Table S3.

### Detection of CF-BD in different *B. dorsalis* populations

To detect CF-BD infection in different *B. dorsalis* populations, 78 flies collected from 13 populations in 2015 (Additional file 7: Table S5) were subjected to real-time quantitative PCR with CF-BD-specific primers in order to obtain the cycle threshold (Ct) value for each fly. A 282-bp region of the *recA* gene in CF-BD was amplified by the specific primers (Additional file 3: Table S3) under a temperature profile of 95 °C for 5 min followed by 35 cycles at 95 °C for 30 s, 55 °C for 30 s and 72 °C for 30 s, and ending at 72 °C for 5 min.

### Statistical analysis

Mortality data for the SS and RS flies were corrected using Abbott’s formula [67], and LC_50_ values were examined with a probit analysis conducted with SPSS (Statistical Package for the Social Sciences) 16.0 software. Variation in the OTU profile similarities was visualized via 2D, non-metric multidimensional scaling (nMDS) plots and was statistically evaluated using nonparametric MANOVA (NPMANOVA). Differences between the treatments and controls were compared with independent-sample *t* tests. Differences were considered significant when the *p* values were <0.05. The data were analyzed using SPSS.

## Additional files

Additional file 1: Table S1. Primer information of the genes. (DOCX 17 kb)
Additional file 2: Table S2. Prevalence of CF-BD in individual field flies. (DOCX 18 kb)
Additional file 3: Table S3. Tag data. (DOCX 17 kb)
Additional file 4: Table S4. Numbers of OTUs in different samples. (DOCX 16 kb)
Additional file 5: Figure S1. Device used for testing the toxicity of trichlorphon to flies. (DOCX 103 kb)
Additional file 6: Supplementary methods. (DOCX 27 kb)
Additional file 7: Table S5. Antibiotic sensitivity of CF-BD. (DOCX 17 kb)
Additional file 8: Table S6. Gene information used for phylogenetic tree construction. (DOCX 20 kb)

## Abbreviations

CF-BD: *Citrobacter* sp.
LC_50_: The median lethal concentration
SS: Resistant strains
SS: Susceptible strains
