# Supplementary material for: Gut symbiont enhances insecticide resistance in a significant pest, the oriental fruit fly Bactrocera dorsalis (Hendel)
Source: Microbiome. 2017 Feb 1;5:13. doi: 10.1186/s40168-017-0236-z (PMC5286733; doi:10.1186/s40168-017-0236-z)
Supplement: Additional file 1: Table S1. — Primer information of the genes. (DOCX 17 kb) [file 40168_2017_236_MOESM1_ESM.docx]

Table S1 Primer information of the genes

| Gene name | Primer sequence | Product size (bp) |
| --- | --- | --- |
| 16S rDNA | F: CACGGATCCAGAGTTTGAT(C/T)(A/C)TGGCTCAG  R: GTGAAGCTTAGGG(C/T)TACCTTGTTACGACTT | 1521 |
| recA | F: GGGCCGTATCGTCGAAATCT  R: GTCAATGCGGCAACAGAGTC | 282 |
| 0086 | F:ACTACATGCGAGAGTGGCTG  R:CTGCGCTCTTTCTCCATTGC | 268 |
| 1012 | F:GCGTTATTGATGCCATGCGT  R:GGCCATATCGATGTGCAGGA | 297 |
| 1747 | F:TCTTCGCTATTCCTGACGGC  R:CCAGTTCCTGCTCGCCAATA | 219 |
| 2752 | F:CTGTGGATTGGCGAGGAAGT  R:CCTCAGCCACATTACGCAGA | 250 |
| 4498 | F:TCTCTGCGCATGGTTTCTGT  R:TCTTTTTGCCAGTGCTTGCG | 276 |
