# Supplementary material for: Gut symbiont enhances insecticide resistance in a significant pest, the oriental fruit fly Bactrocera dorsalis (Hendel)
Source: Microbiome. 2017 Feb 1;5:13. doi: 10.1186/s40168-017-0236-z (PMC5286733; doi:10.1186/s40168-017-0236-z)
Supplement: Additional file 2: Table S2. — Prevalence of CF-BD in individual field flies. (DOCX 18 kb) [file 40168_2017_236_MOESM2_ESM.docx]

Table S2 Prevalence of CF-BD in individual field flies

| Sample name | Sample site | CF-BD infection % (positive/total flies) |
| --- | --- | --- |
| GZ | Guangzhou,Guangdong province | 100(6/6) |
| YF | Yunfu, Guangdong province | 100 (6/6) |
| ZJ | Zhangjiang,Guangdong | 100 (6/6) |
| HBI | Hebao island, Guangdong | 100 (6/6) |
| TS | Taishan, Guangdong province | 100 (6/6) |
| WZI | Weizhou island, Guangdong province | 100 (6/6) |
| ZH | Zhuhai, Guangdong province | 100 (6/6) |
| MZ | Mengzi, Yunnan province | 100 (6/6) |
| CD | Chengdu, Sichuang province | 100 (6/6) |
| SY | Sanya, Hainan province | 100 (6/6) |
| NN | Nanning, Guangxi province | 100 (6/6) |
| ZZ1 | Zhengzhou, Henan province | 100 (6/6) |
| ZZ2 | Zhangzhou, Fujian province | 100 (6/6) |
