# Supplementary material for: Gut symbiont enhances insecticide resistance in a significant pest, the oriental fruit fly Bactrocera dorsalis (Hendel)
Source: Microbiome. 2017 Feb 1;5:13. doi: 10.1186/s40168-017-0236-z (PMC5286733; doi:10.1186/s40168-017-0236-z)
Supplement: Additional file 3: Table S3. — Tag data. (DOCX 17 kb) [file 40168_2017_236_MOESM3_ESM.docx]

Table S3 Tag data

| Sample ID | Number | Total length(bp) | Max length(bp) | Min length(bp) | N50(bp) | N90(bp) |
| --- | --- | --- | --- | --- | --- | --- |
| SS1 | 63891 | 29765540 | 488 | 432 | 466 | 466 |
| SS2 | 62262 | 29011484 | 479 | 441 | 466 | 466 |
| SS3 | 38748 | 18051516 | 479 | 435 | 466 | 466 |
| RS1 | 77789 | 36245221 | 488 | 434 | 466 | 466 |
| RS2 | 64783 | 30185780 | 486 | 437 | 466 | 466 |
| RS3 | 74122 | 34538394 | 487 | 440 | 466 | 466 |

SS: sensitive strain; RS: resistant strain.
