# Supplementary material for: Gut symbiont enhances insecticide resistance in a significant pest, the oriental fruit fly Bactrocera dorsalis (Hendel)
Source: Microbiome. 2017 Feb 1;5:13. doi: 10.1186/s40168-017-0236-z (PMC5286733; doi:10.1186/s40168-017-0236-z)
Supplement: Additional file 4: Table S4. — Numbers of OTUs in different samples. (DOCX 16 kb) [file 40168_2017_236_MOESM4_ESM.docx]

Table S4 Numbers of OTUs in different samples

| Sample ID | Threshold | Number of OTUs |
| --- | --- | --- |
| SS1 | 0.03 | 1803 |
| SS2 | 0.03 | 1300 |
| SS3 | 0.03 | 752 |
| RS1 | 0.03 | 1536 |
| RS2 | 0.03 | 1459 |
| RS3 | 0.03 | 1439 |

SS: sensitive strain; RS: resistant strain.
