# Supplementary figures and images for: Gut symbiont enhances insecticide resistance in a significant pest, the oriental fruit fly Bactrocera dorsalis (Hendel)
Source: Microbiome. 2017 Feb 1;5:13. doi: 10.1186/s40168-017-0236-z (PMC5286733; doi:10.1186/s40168-017-0236-z)

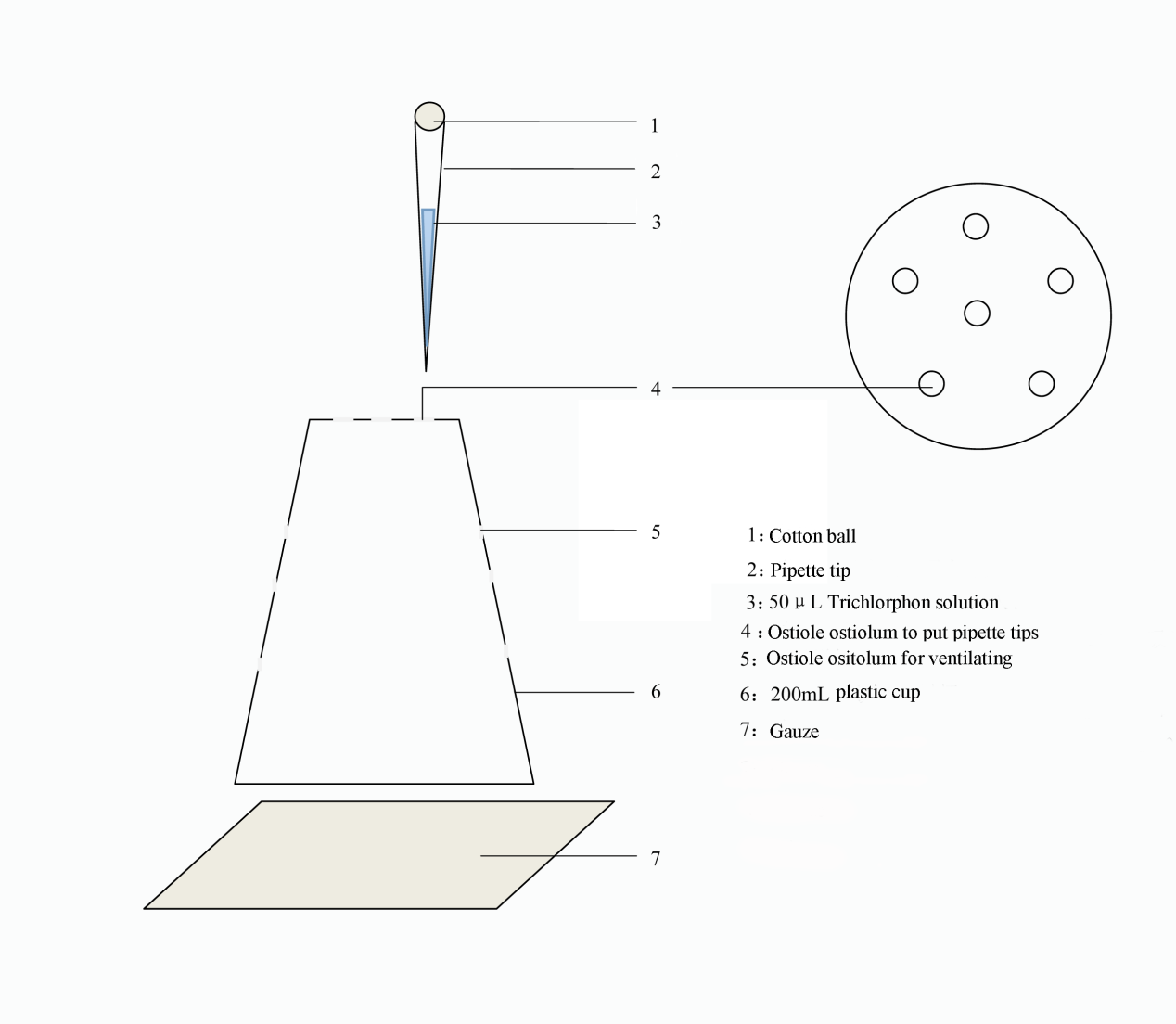


Figure S1 Device used for testing the toxicity of trichlorphon to flies.

Supplement: Additional file 5: Figure S1. — Device used for testing the toxicity of trichlorphon to flies. (DOCX 103 kb) [file 40168_2017_236_MOESM5_ESM.docx]
