# Supplementary material for: Gut symbiont enhances insecticide resistance in a significant pest, the oriental fruit fly Bactrocera dorsalis (Hendel)
Source: Microbiome. 2017 Feb 1;5:13. doi: 10.1186/s40168-017-0236-z (PMC5286733; doi:10.1186/s40168-017-0236-z)
Supplement: Additional file 6: — Supplementary methods. (DOCX 27 kb) [file 40168_2017_236_MOESM6_ESM.docx]

**Supplementary methods**

**V3+V4 region of 16S rDNA amplification and sequencing**

Approximately 465 bp of the V3+V4 region of the bacterial 16S rDNA gene was amplified by PCR according to a standard protocol. The following primers were used: F, CCTACGGGNGGCWGCAG; R, GGACTACHVGGGTATCTAAT. The primers contained the A and B adapters for 454 Life Sciences pyrosequencing, and a unique 12 bp error correcting Golay barcode which allowed multiplexing of samples in one single run. Each strain (RS and SS) was analyzed using three independent replicates in a total reaction volume of 25 μL that contained 2.5 μL Takara 10×Ex Taq buffer, 1.5 μL Mg^2+^ (25 mM), 2 μL dNTP (2.5 mM), 0.25 μL Takara Ex Taq (2.5 U/μL), 0.5 μL each primer (10 µM), 16.75 μL ddH_2_O and 1 μL template. The PCR amplifications were performed with a 2-min incubation at 95°C followed by 30 cycles of 94°C for 30 s, 57°C for 30 s and 72°C for 30 s, and a final 5-min extension at 72°C. The PCR products were purified using QIAGEN MinElute PCR Purification Kit (QIAGEN, Hilden, Germany) to remove unincorporated primers and nucleotides. A micro-spectrophotometer ND-1000 (NanoDrop Technologies, Wilmington, DE, USA) was used to measure the concentration of the purified DNA. The purified DNA was sequenced using the Illumina sequencing kit and an Illumina MiSeq sequencer (Illumina, San Diego, California, USA).

**Filtering of sequence data**

Prior to analyses, the data were filtered to remove low-complexity sequences (such as poly-A sequences) and sequences with ambiguous nucleotides. The filtered sequences were termed target sequences (tags).

**Operational taxonomic unit (OTU) analysis**

To obtain additional information regarding species diversity composition, we subjected the tags to OTU abundance analysis. The number of OTUs was calculated with the Mothur software [[1](#_ENREF_1)] at 97% similarity. Species annotation was also performed where possible. Using the species annotations and the tag number of the OTUs, OTU abundance profiles were generated for all samples. Quantitative similarities of the OTU profiles of SS and RS samples were characterized using the Bray–Curtis similarity index [[2](#_ENREF_2)]. To demonstrate changes in OTU abundance profiles between samples, a heat map was generated with R [[3](#_ENREF_3)]. Because the diversity of OTUs was large and the abundance of some OTUs was low, only those OTUs for which the tags constituted more than 0.01% of the total tags were used to generate the heat map. Based on the species annotation information and the OTU abundance profiles of the different samples, we obtained the OTU abundance for each sample and generated a species-distribution diagram and stack maps with R.

**16S rDNA amplification**

Bacteria were collected from pure cultures for the extraction of genomic DNA using a Bacterial Genome DNA Extraction Kit (Tiangen, Beijing, China) according to the manufacturer’s instructions. 16S rDNA amplification was performed in a total reaction volume of 50 μL with 0.4 μL DNA polymerase (5 U/μL), 5 μL 10× PCR buffer, 4 μL dNTP (2.5 mM), 1 μL each primer (10 µM), 3 μL DNA template and 33.6 μL ddH_2_O. The PCR amplification was performed using an Eppendorf thermal cycler and began with a 5-min incubation at 95°C followed by 35 cycles of 95°C for 1 min, 55°C for 1 min and 72°C for 2 min, and a final extension at 72°C for 10 min. The PCR products were confirmed by electrophoresis in a 0.8% agarose gel and purified with a Gel DNA Mini Purification Kit (Tiangen, Beijing, China). The purified DNA was ligated into the T vector (Tiangen, Beijing, China) and transformed into Top10 *E. coli* cells (Tiangen, Beijing, China) according to the manufacturer’s instructions. The transformed cells were spread on LB agar plates and, after antibiotic selection and blue/white staining, colonies were picked for colony PCR and direct sequencing. The sequences were subjected to a BLAST search against the NCBI database for sequence-homology analysis.

**CF-BD genome sequencing and annotation**

A CF-BD culture isolated from RS flies and grown in BHI media was centrifuged, and cell pellets used for total genomic DNA extraction with a bacterial genome DNA extraction kit (Tiangen, Beijing, China). The quality of the genomic DNA was assessed by gel electrophoresis. For genome sequencing, 5 μg of genomic DNA was used to prepare a shotgun library for Illumina sequencing as recommended by the manufacturer (Illumina, San Diego, CA, USA). The resulting reads were assembled with GS De Novo Assembler version 2.5.3 (Glendale, CA, USA) using default parameters. All obtained DNA sequences were processed in the Consed program, manually inspected, and assembled to generate the complete genome sequence of CF-BD. Initial open reading frame (ORF) identification and annotation were performed by an IMG/ER system [[4](#_ENREF_4)]. Automated gene prediction and annotation of the assembled genome sequence were performed using the locally installed bacterial genome annotation system GenDB v2.2 [[5](#_ENREF_5)], and the phosphate genes were identified. Comparative genomics analyses were performed by applying the BLAST Ring Image Generator [[6](#_ENREF_6)] and Mauve program [[7](#_ENREF_7)] to compare the genome difference between different *Citrobacter* strains. To identify the potential organophosphorus hydrolase genes (OPH), the sequence similarities between the phosphate genes and the OPHs from other bacteria were compared by MEGA5.0 [[8](#_ENREF_8)].

References

1. Schloss PD, Westcott SL, Ryabin T, Hall JR, Hartmann M, Hollister EB, Lesniewski RA, Oakley BB, Parks DH, Robinson CJ *et al*: Introducing mothur: Open-Source, Platform-Independent, Community-Supported Software for Describing and Comparing Microbial Communities. *Applied and Environmental Microbiology* 2009, 75(23):7537-7541.

2. Mccune B, Grace JB: Analysis of ecological communities. *Mjm Software Design Gleneden Beach* 2002, 289(03):448.

3. Kajiwara H, Nakamura M: Hierarchical cluster analyses and heat map analyses of silkworm tissues using R-statistics. *Journal of Electrophoresis* 2008, 52(1):29-38.

4. Markowitz VM, Chen IMA, Palaniappan K, Chu K, Szeto E, Grechkin Y, Ratner A, Jacob B, Huang J, Williams P *et al*: IMG: the Integrated Microbial Genomes database and comparative analysis system. *Nucleic acids research* 2012, 40(Database issue):D115-122.

5. Folker M, Alexander G, Mchardy AC, Daniela B, Thomas B, Burkhard L, Oliver R, Robert G: GenDB--an open source genome annotation system for prokaryote genomes. *Nucleic Acids Research* 2003, 31(8):2187-2195(2189).

6. Alikhan N-F, Petty N, Ben Zakour N, Beatson S: BLAST Ring Image Generator (BRIG): simple prokaryote genome comparisons. *BMC Genomics* 2011, 12(1):1-10.

7. Darling ACE, Mau B, Blattner FR, Perna NT: Mauve: Multiple Alignment of Conserved Genomic Sequence With Rearrangements. *Genome Research* 2004, 14(7):1394-1403.

8. Tamura K: MEGA5: molecular evolutionary genetics analysis using maximum likelihood, evolutionary distance, and macimum parsimony methods. *Mol Biol Evol* 2011, 28:2731-2739.
