# Supplementary material for: Gut symbiont enhances insecticide resistance in a significant pest, the oriental fruit fly Bactrocera dorsalis (Hendel)
Source: Microbiome. 2017 Feb 1;5:13. doi: 10.1186/s40168-017-0236-z (PMC5286733; doi:10.1186/s40168-017-0236-z)
Supplement: Additional file 7: Table S5. — Antibiotic sensitivity of CF-BD. (DOCX 17 kb) [file 40168_2017_236_MOESM7_ESM.docx]

Table S5 Antibiotic sensitivity of CF-BD

| Drug | Diameter of bacterial inhibitory  Rings (mm) | Sensitivity |
| --- | --- | --- |
| Streptomycin | 20±0.54 | S |
| Amikacin | 13±1.01 | R |
| Ampicillin | 7±0.84 | R |
| Sulfisoxazole | 20±0.23 | S |
| Cefazolin | 7±0.58 | R |
| Gentamicin | 18±0.64 | I |
| Sulfamethoxazole | 20±0.78 | S |
| Chloramphenicol | 20±0.59 | S |
| Ciprofloxacin | 28±0.64 | S |
| Tetracycline | 18±0.94 | S |
| Kanamycin | 15±0.89 | I |
| Nalidixic acid | 20±0.98 | S |
| Amoxicillin | 9±0.56 | R |

Notes: “S” sensitive; “R” resistance; “I” intermediate
