# Supplementary material for: Gut symbiont enhances insecticide resistance in a significant pest, the oriental fruit fly Bactrocera dorsalis (Hendel)
Source: Microbiome. 2017 Feb 1;5:13. doi: 10.1186/s40168-017-0236-z (PMC5286733; doi:10.1186/s40168-017-0236-z)
Supplement: Additional file 8: Table S6. — Gene information used for phylogenetic tree construction. (DOCX 20 kb) [file 40168_2017_236_MOESM8_ESM.docx]

Table S6 Gene information used for phylogenetic tree construction

| Gene name | Species | Accession number |
| --- | --- | --- |
| 0085 | CF-BD | KT966406 |
| 0086 | CF-BD | KT966407 |
| 0118 | CF-BD | KT966408 |
| 0176 | CF-BD | KT966409 |
| 0283 | CF-BD | KT966410 |
| 0286 | CF-BD | KT966411 |
| 0307 | CF-BD | KT966412 |
| 0366 | CF-BD | KT966413 |
| 0412 | CF-BD | KT966414 |
| 0476 | CF-BD | KT966415 |
| 0496 | CF-BD | KT966416 |
| 0742 | CF-BD | KT966417 |
| 0785 | CF-BD | KT966418 |
| 1006 | CF-BD | KT966419 |
| 1012 | CF-BD | KT966420 |
| 1165 | CF-BD | KT966421 |
| 1240 | CF-BD | KT966422 |
| 1255 | CF-BD | KT966423 |
| 1412 | CF-BD | KT966424 |
| 1418 | CF-BD | KT966425 |
| 1667 | CF-BD | KT966426 |
| 1683 | CF-BD | KT966427 |
| 1747 | CF-BD | KT966428 |
| 1757 | CF-BD | KT966429 |
| 1803 | CF-BD | KT966430 |
| 1894 | CF-BD | KT966431 |
| 1945 | CF-BD | KT966432 |
| 1967 | CF-BD | KT966433 |
| 2301 | CF-BD | KT966434 |
| 2463 | CF-BD | KT966435 |
| 2627 | CF-BD | KT966436 |
| 2649 | CF-BD | KT966437 |
| 2728 | CF-BD | KT966438 |
| 2752 | CF-BD | KT966439 |
| 2910 | CF-BD | KT966440 |
| 3013 | CF-BD | KT966441 |
| 3243 | CF-BD | KT966442 |
| 3280 | CF-BD | KT966443 |
| 3310 | CF-BD | KT966444 |
| 3406 | CF-BD | KT966445 |
| 3458 | CF-BD | KT966446 |
| 3494 | CF-BD | KT966447 |
| 3683 | CF-BD | KT966448 |
| 3714 | CF-BD | KT966449 |
| 3851 | CF-BD | KT966450 |
| 3866 | CF-BD | KT966451 |
| 3884 | CF-BD | KT966452 |
| 3912 | CF-BD | KT966453 |
| 4015 | CF-BD | KT966454 |
| 4313 | CF-BD | KT966455 |
| 4317 | CF-BD | KT966456 |
| 4389 | CF-BD | KT966457 |
| 4414 | CF-BD | KT966458 |
| 4498 | CF-BD | KT966459 |
| 4692 | CF-BD | KT966460 |
| CAE53631 | *Pseudomonas pseudoalcaligenes* | CAE53631 |
| ACD03602 | *Stenotrophomonas sp. SMSP-1* | ACD03602 |
| AHZ78297 | *Pseudomonas putida* | AHZ78297 |
| AHZ78141 | *Pseudomonas putida* | AHZ78141 |
| AGE84285 | *Cupriavidus taiwanensis* | AGE84285 |
| ADV36778 | *Pseudomonas sp. BF1-3* | ADV36778 |
| ABP65302 | *Burkholderia sp. JBA3* | ABP65302 |
| AKK25265 | *Burkholderia sp. SZL-1* | AKK25265 |
| AAV39527 | *Flavobacterium sp. MTCC 2495* | AAV39527 |
| ACD85809 | *Sphingomonas sp. JK1* | ACD85809 |
| ACU80554 | *Arthrobacter sp. scl-2* | ACU80554 |
| AAT67170 | *Burkholderia sp. FDS-1* | AAT67170 |
| BAA85881 | *Arthrobacter sp.* | BAA85881 |
| BAO35293 | *Serratia marcescens SM39* | BAO35293 |
| AIO33961 | *Burkholderia cenocepacia* | AIO33961 |
| KGC08266 | *Burkholderia multivorans* | KGC08266 |
| KGB93373 | *Burkholderia cepacia* | KGB93373 |
